# A Polymer-Based Capacitive Sensing Array for Normal and Shear Force Measurement

**DOI:** 10.3390/s101110211

**Published:** 2010-11-15

**Authors:** Ming-Yuan Cheng, Chun-Liang Lin, Yu-Tse Lai, Yao-Joe Yang

**Affiliations:** Department of Mechanical Engineering, National Taiwan University, Taipei, Taiwan; E-Mails: d91522008@ntu.edu.tw (M.-Y.C.); gene0921719425@hotmail.com (C.-L.L.); neptune@mems.me.ntu.edu.tw (Y.-T.L.)

**Keywords:** tactile sensing array, shear sensing array, capacitive sensing, micromachining, flexible electronics

## Abstract

In this work, we present the development of a polymer-based capacitive sensing array. The proposed device is capable of measuring normal and shear forces, and can be easily realized by using micromachining techniques and flexible printed circuit board (FPCB) technologies. The sensing array consists of a polydimethlysiloxane (PDMS) structure and a FPCB. Each shear sensing element comprises four capacitive sensing cells arranged in a 2 × 2 array, and each capacitive sensing cell has two sensing electrodes and a common floating electrode. The sensing electrodes as well as the metal interconnect for signal scanning are implemented on the FPCB, while the floating electrodes are patterned on the PDMS structure. This design can effectively reduce the complexity of the capacitive structures, and thus makes the device highly manufacturable. The characteristics of the devices with different dimensions were measured and discussed. A scanning circuit was also designed and implemented. The measured maximum sensitivity is 1.67%/mN. The minimum resolvable force is 26 mN measured by the scanning circuit. The capacitance distributions induced by normal and shear forces were also successfully captured by the sensing array.

## 1. Introduction

In recent years, the development of humanoid robots has progressed rapidly. Intelligent sensing capabilities, such as tactile, temperature, vision and auditory senses, are crucial for humanoid robots to interact with humans and environment effectively and safely. Tactile sensing array are essential for robots to detect physical contact with humans or environment. In addition, for controlling grasping force in minimum, the detection of a slippage between an object and the surfaces of robot-hand/fingers is critical. Therefore, shear-stress sensing capability, which is essential for slippage detection, is desirable for the artificial skins deployed on a robot hand with grasping capability. There were many researches on sensor arrays for normal and shear force detection. In [1–5], silicon-based tactile sensors were realized by using micromachining techniques. In general, silicon-based devices are too brittle to sustain large deformation, so these devices do not have enough flexibility to cover curved surfaces. Therefore, various polymer-based materials, such as parylene, polyimide (PI), or polydimethlysiloxane (PDMS), were proposed as the substrates for flexible sensors or flexible arrays [6–9].

For polymer-based shear-stress sensors, resistive and capacitive sensing mechanisms are frequently used. For the resistive mechanism, sensing elements can be realized by resistive-metal-based materials. Jiang *et al.* [10] created a flexible substrate by spin-coating PI film on a glass wafer. Then polysilicon was doped and patterned as the piezoresistors for sensing the deformation of micromachined membranes. Ascari *et al.* [11] used four piezoresistors embedded in flexible structures as a shear-force sensing element. In [12], flexible shear stress tactile sensor arrays with the piezoresistive cantilever standing vertically in PDMS substrate were presented. Hwang *et al.* [13] proposed a flexible array of tactile sensors, which composes of four metal strain gauges, for measuring normal/shear loads. These resistive-metal-based sensor arrays could give good sensitivities and reliable responses. However, they usually require relatively complex micromachining processes, especially for creating the 2nd metal layer which is essential for matrix scanning.

Shear-stress sensing arrays can also be realized using capacitive sensing mechanisms. Although capacitive sensing may suffer from interference from external noise sources (e.g., in the form of electromagnetic waves), this approach has many advantages, such as linear response, immunity to temperature variations, and highly repeatable responses. Lee *et al.* [14] proposed a capacitive three-axis force image sensor array with excellent spatial resolution. The metal traces (interconnects), which are patterned on flexible substrates (e.g., PDMS or silicon rubber), are relatively long and thin. They are usually vulnerable when the substrate is under large deformation. In [15], a three-axis capacitive tactile sensor, which consists of four capacitors embedded in a rubber substrate, was presented. This approach gives good performance for the applications requiring large covering areas, while its best spatial resolution is about 26 mm. Furthermore, some pressure sensors employ optical fibers as the building blocks of sensing elements [16–18]. These devices might need some bulky and expensive equipment for operations.

In our previous paper, we developed a novel capacitive sensing mechanism to realize a capacitive tactile sensing array with floating electrodes [19]. In this work, as an extension of our previous paper, we present the development of a capacitive *tactile* and *shear stress* sensing array using the same micromachining techniques but with different sensor structure design. The sensing array consists of a micromachined PDMS structure and a flexible printed circuit board (FPCB) layer. Each shear sensing element comprises four capacitive sensing cells arranged in a 2 × 2 array, and each capacitive sensing cell has two sensing electrodes and a common floating electrode. A pillar at the middle of each shear sensing element is designed for shear force detection. The proposed design can effectively reduce the complexity of the capacitor structure without compromise in sensitivity. The force distributions in both normal and shear directions can be captured by the capacitance changes of the cells on sensing elements.

## 2. Design of Skin Structure

**Figure 1.**
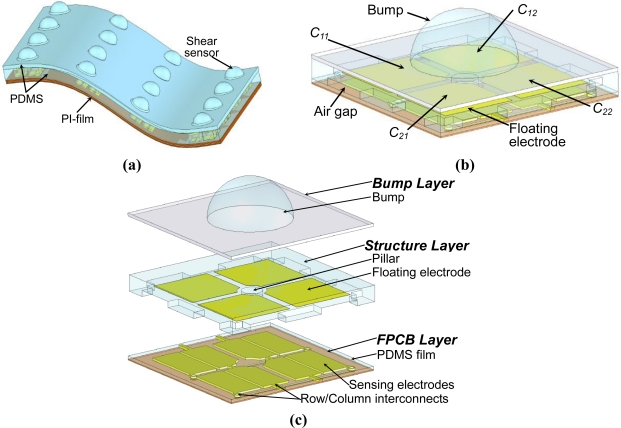
**(a)** The schematic of the artificial skin proposed in this work. **(b)** The magnified schematic of a shear stress sensing element. **(c)** The exploded drawing of the shear stress sensing element.

Figure 1(a) shows the schematic of the proposed capacitive shear stress sensing array. The magnified schematic and the exploded drawing of a shear stress sensing element are shown in Figure 1(b,c). Each sensing element consists of four capacitive tactile sensing cells arranged in 2 × 2 array. A pillar is placed at the center of the four sensing cells [see Figure 1(c)] for ensuring the effectiveness of shear-stress sensing.

**Figure 2.**
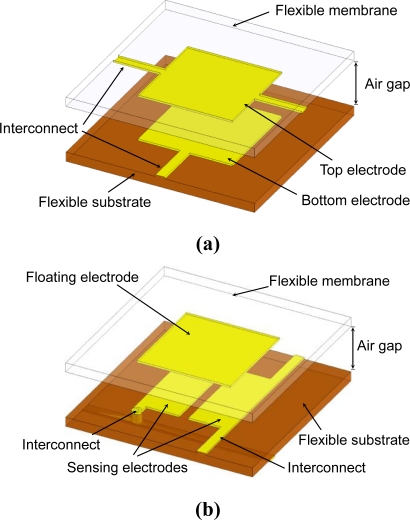
**(a)** The schematic of a typical parallel-plate capacitive mechanism (PCM). **(b)** The schematic of the proposed pseudo-parallel-plate capacitive mechanism (p-PCM).

For the capacitive sensing cells, the most common design is the parallel-plate capacitive mechanism (PCM) [14], as shown in Figure 2(a). However, in this design, long and thin metal interconnect lines (for scanning circuitry) are usually required to be patterned on the top flexible membrane (e.g., PDMS) and on the bottom flexible substrate (e.g., PDMS). These long and thin metal interconnects are quite vulnerable when they are bended to cover curved surfaces. Therefore, in this work, we propose to use the pseudo-parallel-plate capacitive mechanism (p-PCM) [19], as shown in Figure 2(b). In this mechanism, each sensing cell has two sensing electrodes and a common floating electrode. The total capacitance of the sensing cell increases as the floating electrode moves toward the flexible substrate. Also, the total capacitance can be detected by the two sensing electrodes patterned on the substrate. In addition, row and column interconnects for array scanning are also monolithically integrated on the flexible substrate, which can be easily realized by using commercially-available FPCB technologies. FPCBs are widely used in industries and are proved to be quite robust and manufacturable. Furthermore, since all the interconnect lines are located on the flexible substrate (*i.e.*, double-sided FPCB), the scanning circuit can be easily connected to the metal contact pads of the substrate.

**Figure 3.**
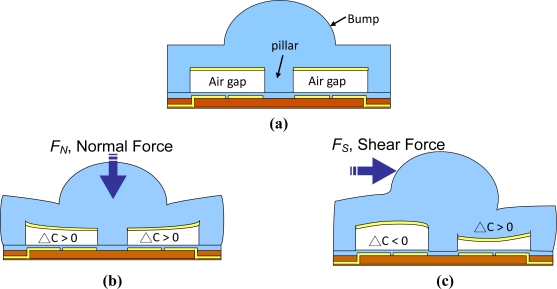
Schematic of a shear stress sensing element **(a)** without applied forces, **(b)** with a normal force, **(c)** with a shear force.

Figure 3 shows the operational principle of the shear stress sensing element. The schematic of the sensing element without external force is shown in Figure 3(a). When a normal force is applied on the bump, the air gap is reduced and the capacitance of each sensing cells increases [Figure 3(b)]. Also, when a shear force is applied on the bump, the air gap on the left side increases whereas the air gap on the right side decreases, as shown in Figure 3(c). The normal force can be estimated by the total capacitance increase of the capacitive sensing cells. The shear force can be estimated by the difference of the capacitance variations between the adjacent sensing cells. Note that the pillar at the middle of the sensing element is critical for shear force detection. A shear force generates a torque around the pillar, so that the capacitance changes of the adjacent sensing cells are in opposite direction.

**Figure 4.**
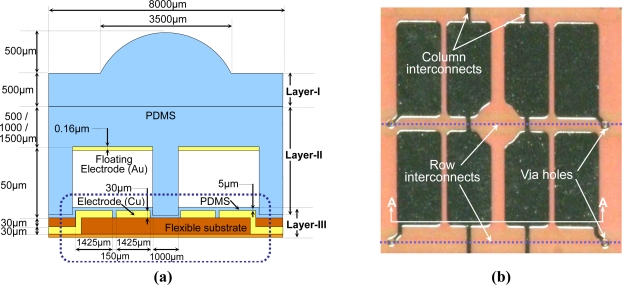
**(a)** The detailed illustration of the proposed shear stress sensing element. This figure is not to scale. **(b)** The picture of a fabricated FPCB layer with four pairs sensing electrodes of a shear stress sensing element.

The detailed structure and dimensions of the proposed shear stress sensing element are shown in Figure 4(a). The sensing element consists of three layers: *the PDMS bump layer* (Layer-I), *the PDMS structure layer* (Layer-II) and *the FPCB layer* (Layer-III). Figure 4(b) is the top view of a fabricated FPCB layer. Note that Layer-III in Figure 4(a) is the cross-sectional view of the $\bar{\text{AA}}$ line in Figure 4(b). The dotted lines indicate the row interconnects on the back side of the FPCB. Floating electrodes are patterned on Layer-II.

## 3. Fabrication of the Device

**Figure 5.**
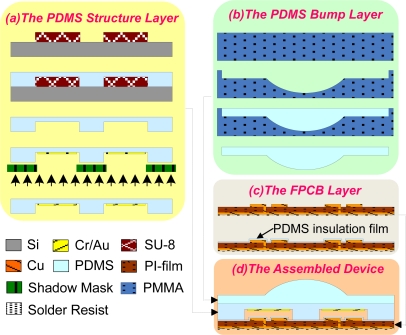
Fabrication process of the shear sensing array.

The process flows of the two PDMS structure layers (Layer-I and Layer-II) and the FPCB layer (Layer-III) are shown in Figure 5. PDMS structure layers are fabricated by the soft lithographic process, which is briefly described as follows: PDMS prepolymer and curing agent (Sylgard 184A and 184B, Dow Corning) are mixed at a 10:1 ratio. After stirring thoroughly and degassing in a vacuum chamber, the prepared PDMS mixture is poured onto a patterned SU-8 master (GM 1070, Gersteltec Sarl). After cured at 90°C for 60 min, the cured PDMS layer is peeled from the master substrate.

As shown in Figure 5(a), chromium (200Å) and gold (1600Å) are deposited on PDMS structure layer as floating electrodes by using an E-beam evaporator. During the evaporation process, a steel shadow mask is placed on the top of PDMS structure layer to pattern the floating electrodes for each sensing cell. Note that the chromium film serves as an adhesion layer. In addition, the PDMS bump layer [see Figure 5(b)] is fabricated by the PMMA mold which is created by using a CNC milling machine. The detailed fabrication process of FPCB can be found in [20]. As shown in Figure 5(c), a *PDMS insulation film*, which serves as insulating layer between floating electrode and sensing electrodes, is formed by spin-coating PDMS (diluted with *n*-hexane at 10:1 ratio) on the FPCB layer. The row and column interconnects are patterned on both sides of the FPCB layer with a total thickness of 100 ìm. The PDMS structure layer and the PDMS bump layer are bonded together after oxygen plasma treatment. Then, the whole PDMS structures are also bonded with the FPCB layer using oxygen plasma treatment. The schematic of the assembled device is shown in Figure 5(d).

**Figure 6.**
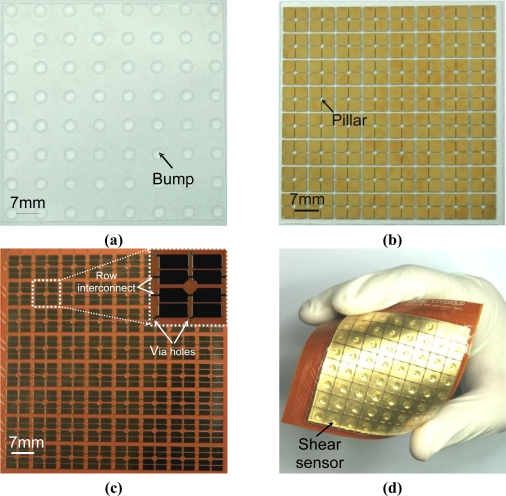
**(a)** The fabricated PDMS bump layer. **(b)** The fabricated PDMS structure layer. **(c)** The FPCB layer. **(d)** The fabricated flexible 8 × 8 shear sensing array.

The fabricated layers are shown in Figure 6. Figure 6(a) is the picture of the PDMS bump layer (Layer-I). Each bump structure is intended to enhance the sensitivity by centralizing the applied force on the top of each sensing element. The dimensions of each bump structure are 3.5 mm in diameter and 0.5 mm in height. The picture of the PDMS structure layer (Layer-II) is shown in Figure 6(b). The deposited metal patterns on this layer are large square shapes without connecting to each other. Therefore, even though the PDMS layer is under a very large force (*i.e.*, under very large deformation or bending), these metal patterns are immune from damage since vulnerable metal traces (long metal lines) do not exist. The pillar of each shear-stress sensing element is also formed on the PDMS structure layer [Figure 6(b)]. The fabricated FPCB layer (Layer-III) is shown in Figure 6(c). The sensing electrode pairs, the via holes and the row interconnects on the PI film are also indicated in the figure. Figure 6(d) shows the picture of the fabricated 8 × 8 shear sensing array. The size of the array is 64 × 64 mm^2^.

## 4. Measurement and Discussion

**Figure 7.**
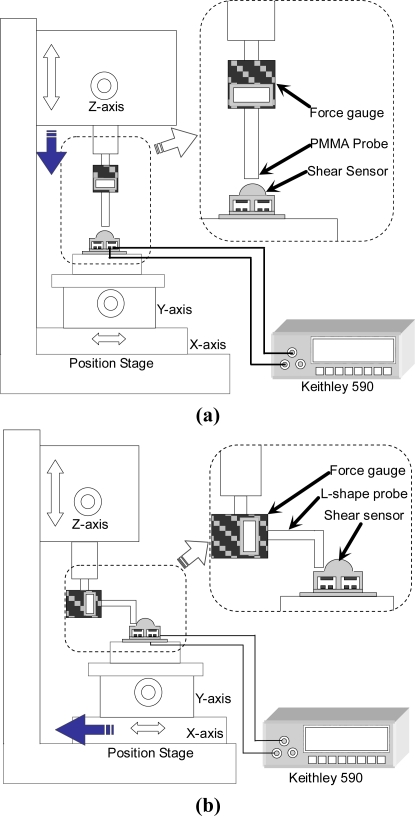
Experimental setup for measuring the capacitance of shear sensor elements with **(a)** applied normal force and **(b)** applied shear force.

Figure 7(a) and 7(b) are the schematics of the experimental setup for measuring normal force and shear force, respectively. A force gauge (HF-1, ALGOL Engineering Co.), whose maximum resolution is 1 mN, is used to measure the applied force. The force gauge is fixed on a z-axis (vertical) translational stage whose displacement resolution is 1μm. The capacitances of the sensing element are measured by a CV analyzer (Keithley 590, Keithley Instruments Inc.). For normal force measurement [Figure 7(a)], a straight PMMA rod is connected to the force gauge. As the z-axis stage table moves down, the bump of the sensing element is pushed by the PMMA rod. For shear force measurement [Figure 7(b)], an L-shaped PMMA rod [Figure 7(b)] is connected to the force gauge. As the x-y stage table moves laterally, the bump of the sensing element is pushed by the L-shaped rod.

In order to study the sensitivities, we designed and fabricated three types of devices with different Layer-II thicknesses. The detailed dimensions of these devices (Device-A, Device-B and Device-C) are listed in Table 1.

**Figure 8.**
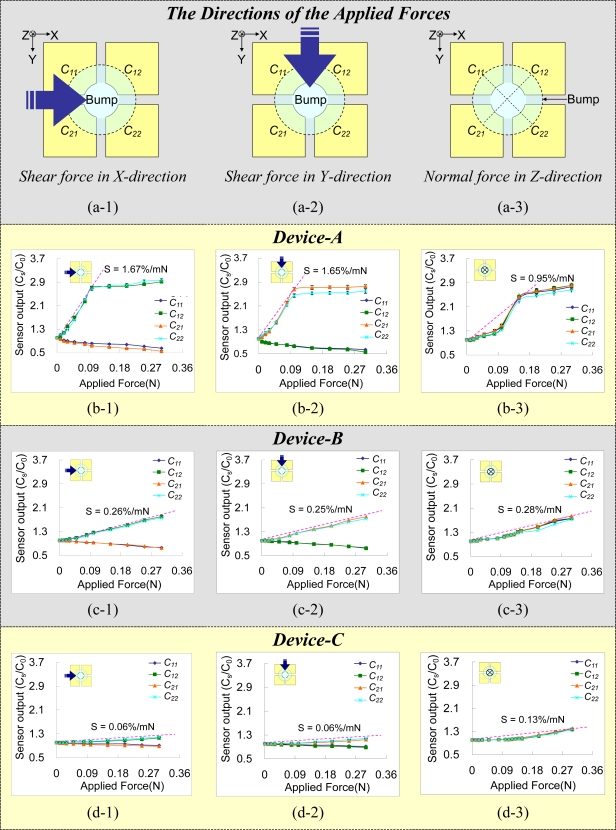
The measured relationships of capacitance *vs.* applied force (N) for three sensing elements with different thicknesses of Layer-II (Device-A, Device-B and Device-C). **(a)** The directions of the applied forces. **(b)** The measured results for Device-A. **(c)** The measured results for Device-B. **(d)** The measured results for Device-C.

Figure 8 shows the measured results of the devices. Figures 8(a-1)∼8(a-3) indicate the directions of the applied forces (*i.e.*, normal forces or shear forces). Figures 8(b), 8(c) and 8(d) are the measured results for Device-A, Device-B and Device-C. The sub-figures in Figures 8(b)∼8(d) are the measured capacitances (*C*_11_, *C*_12_, *C*_21_, and *C*_22_) *vs.* applied forces (N) in the directions indicated in Figure 8(a). For each measured point, the displacement of a stage slowly increases until the force (measured by the force gauge) applied on the sensing element reaches to certain predefined value, and then the capacitance is measured by the CA analyzer. The y-axis of Figure 8 represents the ratio of the measured capacitance to its initial capacitance (the normalized capacitance). Table 2 lists the initial capacitances (*i.e.*, the capacitance under zero external forces) of the four sensing cells of Device-A, Device-B and Device-C. Each data point in Figure 8 is the average result by measuring a sensing element 6 times. The error bars indicate the measured maximum and minimum values. The repeatabilities of these fabricated devices are higher than 92.5%.

Figures 8(b-1), 8(c-1) and 8(d-1) show the results when a shear force is applied in the x-direction. For this configuration, a torque is induced around the pillar of the sensing element, so the capacitances of two sensing cells (*C*_12_ and *C*_22_) increase, while those of the other two (*C*_11_and *C*_21_) decrease. Furthermore, the thickness of Layer-II of Device-A is thinner than those of Device-B and Device-C, so the mechanical stiffness of Device-A is the smallest. Therefore, the measured capacitance of Device-A start to saturate when the applied force reach certain *threshold* values (about 0.1N), as shown in Figure 8(b-1). The threshold value can also be considered as the working range of Device-A. This phenomenon indicates that the floating electrode of the PDMS structure layer almost completely contacts the thin PDMS insulation film coated on the FPCB layer. The results in Figures 8(b-2), 8(c-2) and 8(d-2) are quite similar to those of Figures 8(b-1), 8(c-1) and 8(d-1). Figures 8(b-3), 8(c-3) and 8(d-3) show the results when a normal force is applied in the z-direction. The figure also presents the average sensitivities of the devices, which are defined as the slopes of the linear ranges of these curves [14]. The sensitivities can be increased by reducing the thickness of Layer-II (*i.e.*, reducing the stiffness of the sensing element).

**Figure 9.**
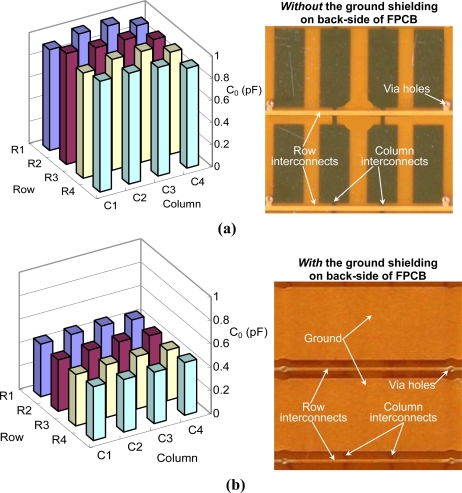
The measured initial capacitances of the sensing mechanisms **(a)** without the ground shielding on the back-side of layer-III, and **(b)** with the ground shielding on the back-side of layer-III.

**Figure 10.**
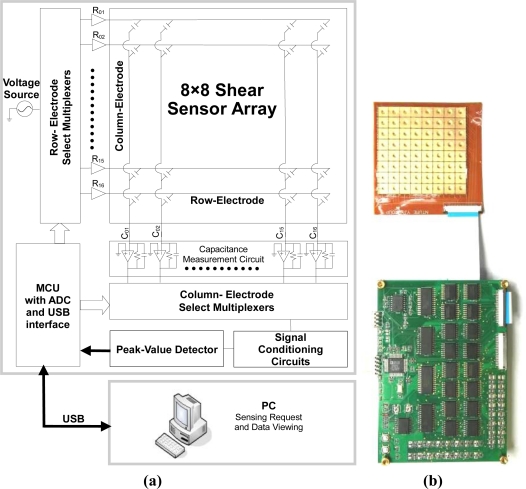
**(a)** The schematic of the sensing-array system with scanning circuitry. **(b)** The picture of the sensing-array with scanning circuitry.

It is worth mentioning that a ground shielding on the back side of the FPCB (layer-III) is also implemented to minimize the capacitance noise level, Figure 9(a) shows the measured initial capacitances of 4 × 4 sensing cells (Device-A) *without* ground shielding. Figure 9(b) is the measured results of the same device with ground shielding. Without ground shielding, the measured average initial capacitances is 965 fF. *With* ground shielding, the measured average initial capacitances is 456 fF, which is quite close to the theoretical value (*i.e.*, the sum of the sensing cell capacitance and the parasitic capacitance). Therefore, the ground shielding can effectively reduces the noise level, which in turn enhances the signal-to-noise ratio of sensing elements. Figure 10(a) is the schematic of the sensing array system, which includes a capacitive sensing array and a scanning circuitry. Multiplexers are used for row and column scanning. The capacitance of a target sensing cell is measured by a simple charge amplifier. The average power consumption of the sensing-array system is about 600 mW.

**Figure 11.**
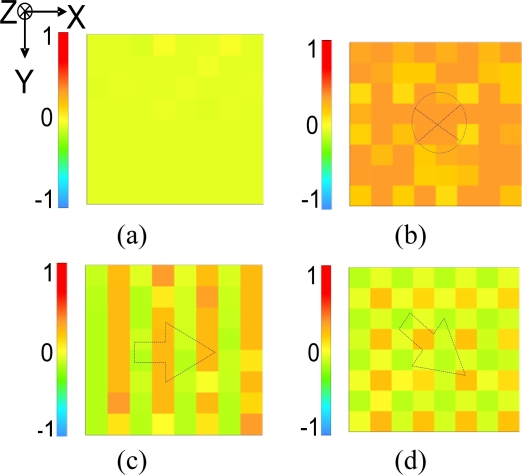
The normal and shear force images captured by a 4 × 4 shear sensing array **(a)** under zero external force, **(b)** under a normal force in z-direction, **(c)** under a shear force in x-direction, and **(d)** under a shear force whose direction is 45 degree with respect to x-axis.

The measured capacitance distributions induced by applying normal and shear forces are also successfully captured by a fabricated 4 × 4 shear stress sensing array (Device-A), as shown in Figure 11. The images are obtained by applying forces on a glass plate which is placed on the top of the 4 × 4 array. The direction of the applied force is shown on the center of the corresponding image in the figure. For Figure 11(b,c,d), the forces applied on the glass plate are 5.5 N, 3.1 N, and 2.2 N, respectively. Also, Figure 11(a) is the capacitance image under zero forces. The measured characteristics of these devices are also summarized in Table 1. Device-A gives the minimum resolvable force (26 mN, measured by the scanning circuit) and the maximum sensitivity (1.67%/mN).

## 5. Conclusions

The development of a capacitive shear stress sensing array was presented in this paper. The proposed sensing array, which employs a novel capacitive sensing mechanism, consists of a micromachined PDMS structure and a flexible printed circuit board (FPCB). Each shear sensing element consists of four capacitive sensing cells. The capacitive sensing cell also comprises two sensing electrodes and a common floating electrode. The design can effectively reduce the complexity of device structure and thus makes the device highly manufacturable. The corresponding scanning circuit was designed and implemented. The characteristics of the devices with different dimensions were measured and discussed. The measured maximum sensitivity is 1.67%/mN. The minimum resolvable force is about 26 mN when measured by the scanning circuit. The measured capacitance distributions induced by applying normal and shear forces are also successfully detected.

## Figures and Tables

**Table 1.** The dimensions and performance characteristics of the fabricated shear sensing elements.

Device	Device-A	Device-B	Device-C
Item			
The size of sensing element (mm^2^)	8 × 8	8 × 8	8 × 8
The size of floating electrode (mm^2^)	3 × 3	3 × 3	3 × 3
The thickness of membrane (mm)	***0.5***	***1.0***	***1.5***
Normal stress sensitivity (%/mN)	0.95	0.28	0.13
Shear stress sensitivity (%/mN)	1.67	0.26	0.06
The ranges of applied force (mN)	0 ∼ 108	0 ∼ 5 05	0 ∼ 812
The force resolution using scanning circuit (mN)	26	151	563

**Table 2.** The initial capacitances (*C*_0_) of four capacitive sensing cells of Device-A, Device-B and Device-C.

Device	Device-A	Device-B	Device-C
*C*_0_			
*C*_11_ (fF)	440	443	485
*C*_12_ (fF)	473	466	447
*C*_21_ (fF)	463	483	477
*C*_22_ (fF)	451	460	455
